# Xeroderma pigmentosum-Cockayne syndrome complex

**DOI:** 10.1186/s13023-017-0616-2

**Published:** 2017-04-04

**Authors:** Valerie Natale, Hayley Raquer

**Affiliations:** Forgotten Diseases Research Foundation, Santa Clara, CA 95050 USA

**Keywords:** Xeroderma pigmentosum, Cockayne syndrome, DNA repair demyelinating disease (CNS), Leukodystrophies, Developmental disorders

## Abstract

Xeroderma pigmentosum-Cockayne syndrome complex is a very rare multisystem degenerative disorder (Orpha: 220295; OMIM: 278730, 278760, 278780, 610651). Published information on XP-CS is mostly scattered throughout the literature. We compiled statistics related to symptom prevalence in XP-CS and have written a clinical description of the syndrome. We also drew on clinical practices used in XP and in Cockayne syndrome without XP to aid management of XP-CS.

Extensive searches of the literature identified 43 XP-CS patients. The diagnosis had been confirmed with molecular or biochemical methods in 42 of them. Clinical features of each patient were summarized in spreadsheets and summary statistics were generated from this data. XP patients are classified into complementation groups according to the gene that is mutated. There are four groups in XP-CS, and classification was available for 42 patients. Twenty-one were in the XP-G complementation group, 13 in XP-D, 5 in XP-B, and 3 in XP-F. Overall, the clinical features of XP-CS are very similar to those of CS without XP, with the exception of skin cancers in XP-CS. However, one intriguing finding was that cancer incidence was lower in XP-CS compared to XP alone or XP-neurological disorder. The cancer rate in XP-CS was higher than in CS without XP, an unsurprising finding. There is preliminary evidence for the existence of severity groups in XP-CS, as is the case in CS.

Although health problems in XP-CS vary both in severity and in when they the first occur, there was overall homogeneity between all complementation groups and putative severity groups. Severely affected patients met fewer milestones and died at younger ages compared to more mildly affected patients.

**Electronic supplementary material:**

The online version of this article (doi:10.1186/s13023-017-0616-2) contains supplementary material, which is available to authorized users.

## Background

### Definition

Xeroderma pigmentosum-Cockayne syndrome complex (XP-CS) is a very rare neurodegenerative disorder that combines clinical features of xeroderma pigmentosum (XP) with those of Cockayne syndrome (CS).

CS is an autosomal recessive multi-system degenerative disorder. It is characterized by photosensitivity, neurodegeneration, intellectual disability, joint contractures that may be severe enough to cause dislocations, hearing loss, and a variety of other problems [1–3]. Life expectancy is affected, with more severely affected patients dying younger. Average age at death has been estimated at 5, 16, and 31 years in severely, moderately, and mildly affected patients respectively [2]. CS is associated with mutations in the genes *CSA/ERCC8* or *CSB/ERCC6*, which have roles in DNA repair. In spite of impairments to DNA repair, CS patients do not develop cancer.

XP is also autosomal recessive and, like CS, impairs DNA repair. Lesions caused by UV light are an important type of damage that cannot be repaired efficiently or at all in both conditions. XP is associated with one of several genes: *XPA*, *XPB/ERCC3*, *XPC*, *XPD/ERCC2*, *XPE/DDB2*, *XPF/ERCC4*, *XPG/ERCC5*, and *XPV/POLH*. Patients are classified into complementation groups based on gene mutation (XP-A, etc.). Patients in groups XP-A to XP-G have defects in nucleotide excision repair (NER). XP-V patients have normal NER, but have a deficiency in the ability to allow DNA replication past unrepaired UV lesions.

Because NER is required for repairing certain types of UV light-induced DNA lesions, photosensitivity is a cardinal feature of XP. XP patients in complementation groups XP-A,-B, -D, -F, and -G develop slow-healing sunburns after brief sun exposure — even on a cloudy day [4]. In this group, the first severe sunburn generally occurs very early in life (often after minimal exposure), and the burn may be mistaken for abuse. Patients in complementation groups XP-C, -E, and -V do not sunburn easily, but still have an underlying impairment of DNA repair [4].

XP patients in any complementation group who are not protected from UV light from an early age develop photodamage that can be severe (e.g., disfiguring solar lentigines and skin atrophy) as well as vision impairment that may include blindness. Damage is permanent. XP patients are also at very high risk for skin cancers. Estimates place this risk at 10,000 times that of the unaffected population for non-melanomas and >2,000 times normal for melanoma [5]. Cancers develop at a median age of 9 years (first non-melanoma) and 22 years (first melanoma) [5].

Roughly one-fourth of all XP patients develop neurological problems, with incidence being higher in certain complementation groups, including those that are prevalent in Japan [5, 6]. Neurological forms of XP can be classified into three relatively distinct groups: XP neurologic disease, XP with trichothiodystrophy (XP-TTD), and xeroderma pigmentosum-Cockayne syndrome complex (XP-CS).

Clinically, there are similarities between these conditions. They include progressive loss of cognitive and motor skills and hearing loss. Differences include hair and nail abnormalities in XP-TTD and progeria in XP-CS. Additionally, XP neurologic disease results from primary neurodegeneration, while XP-TTD and XP-CS do not [7]. XP-CS patients have a form of leukodystrophy called *tigroid demyelination*. This term refers to an abnormality that can be seen on T2-weighted MRI scans, in which small patches of preserved myelin (generally around blood vessels) occur within demyelinated areas [8, 9]. Tigroid demyelination also occurs in CS patients without XP (referred to as simply *CS* hereafter) [10, 11].

XP-CS is essentially a combination of XP and CS. Overall, patients have cutaneous features of XP and follow a trajectory that mirrors that of Cockayne syndrome: growth and acquisition of new skills is followed by a plateau period, which is then followed by a period of decline. In CS, the ﻿duration﻿ of these periods depends on disease severity. The most severely affected patients grow and develop the least and reach a stage of decline early (before age 5). Many in this group will not learn to walk or talk. Patients who are very mildly affected may not begin to decline until their teens or later, with decline occurring more slowly and lasting longer. These individuals may learn to read, write, swim, ride a bike, and/or ski [2].

Regardless of disease severity, all CS and XP-CS patients have the same overall medical problems. The primary difference between severity groups relates to timing and severity. Thus, for example, microcephaly is nearly universal in CS, yet may vary from several standard deviations below the mean for age to roughly two standard deviations below it. Similarly, nearly all patients with CS or XP-CS have short stature, but the most severely affected patients are the smallest, and the mildly affected ones are the largest.

XP-CS is very rare. To date, the largest summaries of XP-CS cases had data on only 9 individuals [8, 9]. The aim of this study was to examine all cases of XP-CS reported in the literature to date. We identified 43 cases in the literature between 1965 and 2017, a span of 52 years.

## Methods

The protocol for this study has been registered with PROSPERO (#CRD42016044112).

### Literature search

Our overall strategy was to find cases of XP-CS, which are due to mutations in *XPB*, *XPD*, *XPF*, and *XPG*. We searched PubMed, SciELO, Hindawi, the African Journal Archive, and Google Scholar between January and August 2016. We also scanned publication references, ‘cited by’ papers in Google Scholar, and OMIM entries. Patients with mutations in *ERCC1* were not included. A final search was performed immediately before publication. Search terms were “XP-CS,” “xeroderma AND Cockayne,” “xeroderma AND COFS,” “‘De sanctis’ AND xeroderma” and “XP < −letter > patient.” The majority of searches were in English. However, we also searched in French, German, and Spanish. We also contacted researchers to ask questions about information in publications.

### Study selection and data extraction

Publications selected for review had abstracts describing persons with XP and a neurological disorder. Publications were read carefully to determine if the patient had XP-CS. Of 43 individuals included here, (Additional file 1: Table S1 and Additional file 2: Bibliography 1), 42 had been diagnosed with XP-CS via molecular or biochemical methods. The published clinical features of the sole case without a laboratory diagnosis fit the description of XP-CS very well [12]. Communication with the authors supported this impression. That case was therefore included. Overall, selection was deliberately stringent to ensure that we analyzed only XP-CS patients. Note that a 44^th^ patient, XP40GO (XP-G), may have had XP-CS, but phenotype was not confirmed, as no clinical information was available [13].

### Statistical analysis

Most data here is reported as the number of patients positive for a trait out of the total in whom it was reported as present or absent. When comparisons between XP-CS and other forms of XP were made, a Fisher’s exact test was used to determine significance.

### Findings

#### Demographics

Patient sex was known in 41 cases (19 females, 22 males). Patients or their parents were from 18 countries on 4 continents (Africa, Asia, Europe, and North America; Additional file 1: Table S2). Japan and the USA had the most patients (6 each), followed by the UK (5), and Germany (4). Consanguinity between parents was relatively common, occurring in 8/29 cases. Overall, these statistics are similar to those for XP patients as a whole, which indicate that XP occurs in all ethnic and racial groups [6]. The same is true of CS.

#### Complementation groups

**Fig. 1:**
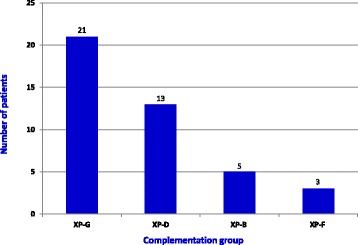
Complementation groups in XP-CS. Complementation group information was available for all but one patient (total: 42 patients). Half of patients with known mutations were in the XP-G group. *XPD* mutations occurred in nearly 1/3 of patients (31%)

There are four complementation groups in XP-CS: XP-B, -D, -F, and -G. Complementation group information was available for 42 patients: 81% belonged to groups XP-G and -D (Fig. 1). All 6 Japanese patients were in the XP-D group, and all 5 Arab patients were in the XP-G group, as were the 3 African or African-American patients (Additional file 1: Table S3). Caucasians bore mutations in all four genes, but all five cases of *XPB*-CS were in this group.

Extensive literature searching identified 42 XP-G patients with any form of XP (XP only, XP-CS, etc.; see Additional file 3: Bibliography 2 for XP-G and -F patients without CS). Thus, the 21 XP-G-CS patients comprised 50% of known XP-G patients. Eight additional XP-G patients had XP-neurologic disease or neurological symptoms, meaning that neurological problems occurred in 69% of XP-G patients.

Neurological problems developed in adulthood in some XP-G patients, such as at age ~58 in patient XP101BR [14], and the mid-thirties in two others [14]. Alternatively, 3 Japanese patients were aged 32, 40, and 60 without neurological problems (XP31KO, XP3HM, and XP52HM, respectively) [15–17]. None of these patients had what could be considered XP-CS.

A 2006 study listed 9 XP-B patients: 5 had XP-CS, 2 had XP-TTD, and 2 had XP with mild neurological problems [18]. We identified one additional XP-B patient: XP84BR, with minimal neurological abnormalities at age 35 [14]. Thus, half of this small group had XP-CS, and all or nearly all had neurological problems.

Analyses of the XPB-CS patients indicated that they had a relatively mild form of XP-CS [8, 9]. However, more patients are needed to determine if this situation was coincidental or a true reflection of XPB-CS.

It was not possible to determine the percentage of XP-D patients with CS due to the very large number of XP-D patients reported in the literature. However, given that only 13 XP-D-CS patients were identified out of a likely total of 100–200 XP-D patients (author’s estimate), XP-CS is relatively rare in this group.

As with XP-G patients, some XP-D patients develop neurological abnormalities as adults (patients XP62TA, age 54; XP29TA, age 45; and XP59BR, age ~50) [14, 19], while some still had not by middle age (e.g., XPD2KO, age 51; XPD3KO, age 48; and XPD6KO, age 61) [20]. Again, however, no XP-D patient has been reported as developing signs of XP-CS in adulthood.

Disease severity varied in the XP-F/CS patients. One (CS1USAU) [21] has mild XP and mild CS. XFE/XP51RO survived until age 16 and was described as meeting early developmental milestones [22]. This characteristic may occur in moderately or mildly affected CS patients, but not in severely affected ones [2]. Patient XPCS1CD suffered global developmental delay with severe growth retardation. She survived until age 12. She had features of XP-CS as well as Fanconi anemia [21].

Patient XFE was described as having a new syndrome, *XFE progeroid syndrome* [22]. This diagnosis was based on apparent clinical inconsistency with XP, CS, XP neurologic disease, and XP-CS. However, his clinical features overlapped closely with those of XP-CS patients. For example, his liver disease, kidney disease, and hypertension were noted as being inconsistent with XP-CS or CS. However, a number of XP-CS patients have had these problems [2, 21, 23–27]. This group includes two patients with mutations in *XPF* (CS1USAU & XPCS1CD). These three problems are common in Cockayne syndrome [1–3].

Additionally, many of *XFE’s* laboratory test results and clinical features were consistent with XP-CS (e.g., DNA repair profile, hearing loss, optic nerve atrophy, a high-pitched voice, ataxia, delayed pubertal development). In addition, his facial features as described and as evident in photographs were consistent with a CS phenotype. Taken together, his overall clinical picture was suggestive of XP-CS, and he was therefore included in this survey.

#### Development of CS signs in adulthood

Signs of Cockayne syndrome have been reported as developing in at least one adult with mutations in *CSB* [28]. We examined the literature to determine if any patients with XP mutations developed signs of CS.

XP24BR, an XP-F patient in her 40s, developed XP-CS-like clinical features, including deep-set eyes, a prominent nose, hearing loss, ataxia, and cognitive decline [14]. However, many of these features overlap with XP-neurological disease, and her condition is therefore not fully consistent with XP-CS at this time (personal communication from authors).

Two adult patients in Israel developed a syndrome that was similar to XP-CS. They were CO14TA, a 54 year-old female and CO107TA, her brother, who died at age 49. Both patients had mutations in *XPF/ERCC4*. They began to develop CS-like facial features in their 20s, and MRI scans showed myelin abnormalities. Neither patient suffered from intellectual disability — both were employed in clerical positions. However, they did experience behavioral changes, including aggressive behavior (H. Slor, personal communication and reference [29]).

XP24BR, CO14TA, and CO107TA all shared a common mutation that leads to the protein change Arg799 > Trp. This mutation is relatively common in XP-F patients, and most patients harboring it develop symptoms of neurodegeneration as children or adults [30]. One patient with this mutation (XP26BR) was reported as not having signs of neurodegeneration [30, 31], but the patient’s age was not given. In XP-F adults, neurodegeneration does not necessarily resemble CS.

No other patients with XP mutations have developed CS signs in adulthood.

#### Clinical features of XP-CS

XP-CS is a multisystem progressive disorder, and neurological problems were progressive in all patients for whom information was available (*n* = 26).

Table 1 shows the frequency of neurological abnormalities in XP-CS patients. All problems listed in this table also occur in CS without XP, in roughly the same percentages. In some cases (e.g., seizures), the value in Table 1 may be an overestimate, as some authors may not have noted the absence of the problem.

**Table 1:** Neurological abnormalities in XP-CS patients

Clinical feature	#/total (%)
Intellectual disability	38/38 (100)
Progressive neurological problems	26/26 (100)
Ataxia, any form (sometimes unspecified)	15/15 (100)
Microcephaly	30/32 (94)
Abnormalities of myelination (CNS)	14/15 (93)
Brain atrophy or ventriculomegaly	19/21 (90)
Hearing loss (sensorineural)	18/21 (86)
Slowed nerve conduction velocity	12/14 (86)
Seizures	8/11 (73)
Brain calcifications	10/16 (63)
Lost or reduced deep tendon reflexes	3/11 (27)

Common imaging findings in XP-CS included intracranial calcifications, brain atrophy, and tigroid demyelination. These findings are hallmark features of CS. Calcifications may not be present in very young children, but may develop later [10]. Little work has been published on the significance of calcifications in CS or XP-CS, and one study of 19 CS patients found no strict correlation between calcifications and patient age, severity of neurological problems, or extent of cerebral atrophy [10].

Hearing loss occurred in 86% of 21 patients. This problem appears to occur universally in CS patients as the syndrome progresses, and can have very serious effects on a quality of life [2]. Patients who lose their hearing may feel isolated and become withdrawn and/or depressed. These factors can accelerate decline. Cochlear implants are commonly used to address this problem, with subjective improvement in quality of life reported﻿ (author's personal knowledge and reference [32]). Some parents of patients with CS have had the implants placed before hearing loss became serious, as a way of avoiding this problem in the first place.

Hand tremors were reported in 7 patients, but their absence was not noted. Tremors are common in CS, and treatment with carbidopa-levodopa appears to reduce their severity and improve fine motor skills [33].

Although we lacked sufficient data to analyze dysarthria, it appears to be common in XP-CS (as it is in CS). Lack of data was mostly due to the large number of XP-CS patients who did not acquire speech.

#### Growth and development

Very short stature was nearly universal (33/34 patients; Table 2). As in CS, height in XP-CS is often three or more standard deviations below the mean for age. Growth hormone levels have been measured as abnormal (high or low) in a small number of CS patients, but have been normal in the majority of patients tested (reviewed in [1]). Treatment with growth hormones had no effect in a single patient in 1958 [34], and their use in CS (and possibly XP-CS by extension) is not recommended due to potential for tumorigenesis [35].

**Table 2:** Developmental and ophthalmological features in XP-CS

Growth and development	#/total (percent)
Intellectual disability	38/38 (100)
Stature ≤3^rd^ percentile	33/34 (97)
Delayed speech development	18/19 (95)
Delayed or absent development of motor skills	25/28 (89)
Failure to thrive	28/32 (88)
Cryptorchidism	9/11 (82)
Hypogonadism (male or female)	8/11 (73)
Low birthweight	14/20 (70)
Preterm birth (gestational age <37 completed weeks)	3/14 (21)
Ophthalmological	
Sunken/deep-set eyes	24/26 (92)
Microphthalmia	10/14 (71)
Pigmentary retinopathy	12/18 (67)
Cataracts	9/20 (45)

Weight in XP-CS patients tends to be very low, even as a function of height, and poor weight gain may be exacerbated by vomiting and/or acid reflux. Use of feeding tubes, such as a jejunal tube (j-tube) may alleviate this problem. J-tubes alleviate vomiting and reflux. An additional advantage of tubes is that they allow easy delivery of many medicines, as well as accurate dosing.

Importantly, CS patients do not appear to have the caloric needs expected for age or body size. This trait has been noted in the literature [36] and in medical records of 3 CS patients (author’s unpublished data). Therefore, a test of resting energy expenditure may be important for determining appropriate caloric needs in XP-CS or CS.

Early developmental delays are universal in all but the mildest cases of CS. In XP-CS, intellectual disability was universal in 38 patients for whom data was available. Microcephaly (94% of patients) was often severe: head circumferences data was provided for 16 patients, and values were at least 5 standard deviations (SDs) below the mean in 15 (the 16^th^ value was–4.1 SDs below the mean). Growth in XP-CS may halt early, such as by 18 months [25]. Extreme microcephaly is also a feature of Cockayne syndrome [37–41], where severity of microcephaly and intellectual disability is correlated with disease severity [2]. There is not enough data to draw a conclusion about this correlation in XP-CS.

Delayed motor skill development was very common in XP-CS patients. The most severely affected individuals did not progress past the level of an infant, with some not passing the developmental level of a two-month-old [23, 25, 42, 43]. Mildly affected patients were larger and able to walk and attend school [21, 23, 44].

#### Ophthalmological manifestations of disease

Table 2 shows ophthalmological manifestations in XP-CS. The most frequent abnormality was deep-set eyes, in 92% of 26 patients. Abnormalities reported in too few patients to analyze included pterygium (1 patient) and photophobia (6 patients).

#### Cutaneous abnormalities and skin cancer

Forty out of 40 patients had lab-confirmed photosensitivity, with the vast majority also sunburning easily (Table 3). The sole patient who did not burn easily was XP56BR (XP-G) [14]; a teenaged boy of Somali origin who may have gained extra protection from having a Fitzpatrick skin phototype of V or VI.

**Table 3:** Skin abnormalities in XP-CS

Skin manifestations	#/total (percent)
Photosensitivity (laboratory measured)^a^	40/40 (100)
Sunburns easily	32/33 (97)
Solar lentigines/abnormal freckling	35/37 (95)
Very dry skin	14/15 (93)
Wizened/progeroid facial appearance	16/23 (70)
Skin cancer	5/33 (15)

^a^Tested in skin fibroblasts as abnormalities after UV irradiation (survival, DNA repair, recovery of RNA synthesis, or unscheduled DNA synthesis)

Progeroid features were noted in 70% of patients. Although commonly associated with both CS and XP-CS, progeria does not tend to occur until the syndrome has progressed. Thus, its absence — especially in a very young person — should not be used to exclude a diagnosis of CS or XP-CS.

**Fig. 2:**
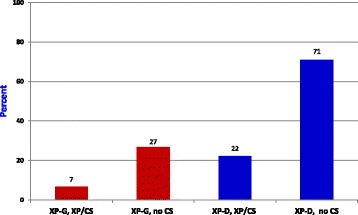
Cancer by complementation group and phenotype. Cancer is less common among XP-CS patients than in the XP population without CS. *Red*: XP-G. *﻿Blue*: XP-D

Skin cancer is very common in XP, but, at 5/33 patients, was not common in XP-CS compared to patients with other forms of XP (Fig. 2). Cancers are not a feature of CS, even in patients who survive into their 30s and beyond (author’s unpublished data and Zhang et al. [45]).

The difference in cancer prevalence between XP-D patients with and without CS was statistically significant according to a Fisher’s exact test (*p* < 0.01). This was not the case for XP-G patients (*p* > 0.05). Similarly, when we compared cancer rates in XP-CS patients and 142 XP-B, -D, -F, and -G patients in the literature, the difference was significant (5/33 XP-CS patients with cancer vs 75/130 non XP-CS patients with cancer; *p* < 0.005).

While reduced life expectancy in XP-CS likely contributes to reduced malignancy rate, three XP-CS cancer patients developed their first malignancies early. XPCS2 and XP1JI (both XP-D) were 2 and 2.5 years old at tumor onset; XPCS4RO (XP-G) was <1 year [23, 46, 47]. In comparison, the youngest age of tumor onset in 4 non-CS XP-G patients was 20 years (XP119BR) [14]. Three other XP-G patients developed cancers at ages 32, ~40, and 54 [15–17], making tumor onset before age 1 in XPCS4RO exceptionally early.

Among 42 XP-D cancer patients without CS, only one had tumor onset at age 2, and the next youngest was 4 (average age: 23 years; data not shown).

The low cancer rate in XP-CS and its absence in CS implies that a biochemical feature of Cockayne syndrome may mitigate cancer risk. At the same time, however, XP-CS patients who do develop cancer may have another trait that makes them prone to developing it early. More data and further studies are needed to confirm or refute this idea. Regardless, avoidance of the sun and UV light is important to minimize cancer risk in XP-CS patients.

Resistance to cancer in CS is not completely understood, but it may be related to an increased tendency toward apoptosis in CS cells [48, 49]. A recent study found that, when comparing to control cells, UVC-induced mutagenesis was not always increased in fibroblast cells from patients with mutations in *CSA* and *CSB*, whereas it was increased in patients with mutations in *XPC* [50]. This finding may help explain lack of cancer in CS patients, but it did not examine cells from XP-CS patients.

In another minor paradox, skin cancer risk in XP-TTD patients is also not higher than the general population’s [51, 52]. This difference may be due to unstable TFIIH proteins not accumulating at the sites of photodamage. TFIIH is a basal transcription factor involved in nucleotide excision repair (NER) and transcription. Lack of NER protein accumulation may allow translesion DNA synthesis, thereby not increasing skin cancer risk [51].

#### Musculoskeletal and other problems

As in CS, joint contractures were very common in XP-CS. They were reported in 17/17 patients for whom data was available. Contractures can hinder mobility, can worsen with time, and may cause joint dislocation. Strategies used for CS patients that may help in managing them in XP-CS include physiotherapy, botox injections, and heel cord lengthening procedures.

Scoliosis was reported in 6 out of 9 XP-CS patients. As noted above, liver disease, kidney disease, and hypertension were also reported (4, 3, and 3 patients, respectively). Kidney disease was the second leading cause of death in a previous survey of CS [2].

Finally, carious teeth, which are a common problem in CS, were reported in 5 out of 8 XP-CS patients. While 8 total patients is too few to draw any statistical conclusions, the number with the problem was high enough that teeth should be monitored carefully in any person with XP-CS. Caries may occur in spite of good oral care [2].

#### Survival in XP-CS

Many XP-CS patients died young. For example, the average at death was 3.7 years for ten XP-G patients (range: 7 months to 6.4 years). However, a further nine XP-G patients were still alive at time of reporting, at an average age of 12 years (range: 5–28 years). Thus, as in CS, there is a variation in life expectancy in XP-CS. In CS, life expectancy is correlated with disease severity, with more severely affected patients dying younger and many mildly affected patients living into their 30s or beyond [2].

All deceased XP-D patients except for one were <14 years old at time of death. The sole outlier was 43 years old [23]. The average age of death of the other patients was 2.9 years. One patient was still alive at time of reporting (XP89MA; age 15; [53, 54]). It is possible that mutations in *XPD* tend to confer a relatively severe phenotype and/or that *XPD*-CS is underdiagnosed in mild cases.

Given the limited data on XP-CS, making estimates of average age of death was not possible, with the exception that *severely affected patients die younger*.

Estimates of life expectancy in CS are most informative when calculated by severity group and may be misleading when calculated otherwise. For example, one review of CS calculated an average age of death of 12 years in 37 patients whose disease severity varied [1]. This figure can cause confusion among caregivers because it does not reflect the fact that many children die at age 5 and others survive into their 20s or later. Surviving to 12 is very rare in severely affected patients, while death at this age would be uncommonly young in mildly affected patients.

Data on age of death in 117 CS patients was used for a previous study [2]. Two calculations were made: mean overall age at death and age at death by severity group. The mean overall age was 11.5 years, yet only 2 patients died at age 11.

Average age of death in three severity groups was calculated as 5, 16, and 31 years [2]. These estimates fit much better with patient survival (16/73 severely affected patients died at age 5, for example). Medians were close to means in these estimates (<1 year for mildly affected patients, ~0.1 year for moderately and severely affected patients).

Thus, average ages of CS patients as a whole do not reflect survival in CS accurately, due to survival differences in severity groups.

#### Clinical differences between XP-D and XP-G patients

We compared XP-D and XP-G patients see if there were phenotypic differences beyond cancer between the two groups. We did not include XP-B and XP-F patients due to low patient numbers. Even with the XP-D and XP-G patients, the data was limited.

Patient numbers were sufficient to make a comparison for a small number of clinical features (stature, failure to thrive, intellectual disability, skin freckling, neurological abnormalities, photosensitivity, propensity to sunburn, presence of sunken eyes, and sex). The percentage of patients with each of these features was essentially identical in both groups. Two possible exceptions were failure to thrive and sex: 10/10 XP-D patients had failure to thrive, compared to 10/12 XP-G patients. This difference was not statistically significant according to a Fisher’s exact test. A check of the author’s CS data (CS-A and CS-B groups) showed that 94% of 44 patients had failure to thrive.

Similarly, only 3 of 12 XP-D-CS patients were female (6 expected for an autosomal recessive condition). Again this figure was not statistically significant, but further studies may determine if XP-CS mutations are more lethal in ﻿embryonic﻿﻿ females.

As more data becomes available on XP-CS patients, phenotypic differences between complementation groups may emerge. For example, we were unable to compare the prevalence of dental caries, seizures, pigmentary retinopathy, or brain calcifications, due to lack of data.

### Diagnostic criteria for XP-CS

The diagnostic criteria for XP-CS are essentially a combination of those for XP and CS, which have been established [35, 55]. The criteria below have been adapted slightly for XP-CS. Features that can help distinguish XP-CS from XP neurologic disease are boldfaced. The Forgotten Diseases Research Foundation has a free online tool for helping to diagnose rare diseases at www.forgottendiseases.org.

### Diagnostic criteria for Cockayne syndrome [35]

#### Major

- Postnatal growth failure (<5th percentile by age 2)
- The following manifest as early developmental delay in most patients:
  - Progressive microcephaly (microcephaly may be present at birth)
  - Neurologic dysfunction
- Progressive deterioration of behavior and intellect (all individuals)
- **Leukodystrophy on brain MRI (characteristic tigroid demyelination as noted in text**; abnormal myelination reported in 93% of patients here)
- **Intracranial calcifications** (may not appear until later; in 63% of patients surveyed here)

#### Minor criteria

- Cutaneous photosensitivity
- Demyelinating peripheral neuropathy diagnosed by electromyography, nerve conduction testing, and/or nerve biopsy (too few XP-CS patients to report here)
- **Pigmentary retinopathy** (67% of patients surveyed here) and/or cataracts (47% of patients surveyed here)
- Sensorineural hearing loss (86% of patients surveyed here; likely universal as the syndrome progresses)
- Dental anomalies, including **carious teeth** (63% of 8 patients surveyed here), enamel hypoplasia, anomalies of tooth number and anomalies of tooth size and shape
- **Cachectic dwarfism** with thinning of the skin and hair, sunken eyes, and a stooped standing posture (Very short stature is universal. Other problems may not appear until the syndrome has progressed; a somewhat CS-like facial appearance may also occur in older adults with XP neurologic disease [56, 57].)
- Characteristic radiographic findings of thickening of the calvarium, sclerotic epiphyses, vertebral and pelvic abnormalities (not surveyed here)

### Diagnostic criteria suggestive of xeroderma pigmentosum [55]

We have omitted the criteria for XP neurologic disease, as neurological abnormalities are described for CS above.

#### Skin

- Acute sun sensitivity (severe sunburn with blistering or persistent redness on minimal sun exposure)
- Solar lentigines/freckling on the face before age two years (occurred in 95% of XP-CS patients, appearance was <2 years if age was reported)
- Skin cancer before age ten (skin cancer is less common in XP-CS patients, but may occur very early in life, as noted in text)

#### Eyes

- Photophobia with prominent conjunctival injection (noted in XP-CS 6 patients, but absence not noted) (The following were not noted in sufficient patients for analysis in our literature review; some XP-CS patients may not live long enough for these problems to become manifest. Many of the features below appeared to occur in at least one adult with XP-CS, patient XP11BE [58].)
- Severe keratitis, which may cause opacity of the cornea and corneal vascularization
- Increased pigmentation of the eyelids with loss of lashes
- Atrophy of the skin of the lids resulting in ectropion, entropion, or in severe cases, complete loss of the lids

Two important points about photosensitivity and progeria must be kept in mind. Photosensitivity, in the form of easy sunburning, may not be evident in a patient (especially an XP-F patient). Its absence should not be used to exclude a diagnosis. Photosensitivity as tested by laboratory UV irradiation is, however, likely universal in XP-CS. Similarly, while progeria is a common feature of XP-CS, it occurs as the syndrome progresses, and is not always present in very young patients. Again, its absence should not be used to exclude a diagnosis of XP-CS.

While there is considerable overlap between XP-CS and XP neurologic disease, the two conditions may be distinguished by the features in boldface text above. In particular, distinctive CS-like facial features tend to appear earlier in more severely affected patients. As noted above, in CS, these patients tend to be smaller and achieve fewer milestones than more mildly affected patients.

Finally, sequencing for mutations in the genes listed in the Introduction can aid diagnosis.

## Discussion

XP-CS is a very rare disorder that combines the clinical features of XP and Cockayne syndrome. Patients are exquisitely sensitive to UV light, and are at increased risk for malignancies and vision loss. They also exhibit growth failure, developmental delays that can be severe, progressive neurological abnormalities, and reduced life expectancy.

The 43 XP-CS case reports analyzed here allow a basic meta-analysis of the features of XP-CS, which was not possible when the last meta-reviews of XP-CS were published [8, 9]. For example, it appears that there are severity groups in XP-CS, as in CS. Using criteria from our earlier study [2], it was possible to make rough severity group assignments for 35 of the patients analyzed here: 22 were severely affected, 4 moderate, and 13 mild. These groupings are rough, but generally, patients with early death and/or poor early development were classified as severe. Those with death after age 25 and/or with good early development were classified as mild. A classification of moderate XP-CS was difficult to make, given that in CS, 1) effects on very early development may not be obvious, and 2) these patients may be almost as small as severely affected patients.

After careful consideration, it was determined that patient XFE’s clinical features match those of XP-CS sufficiently to warrant his inclusion in the XP-CS group. In light of the neurological variation that occurs among all XP patients — and particularly given new information about phenotypes in XP-F patients — defining a new disease based on a single patient whose clinical features overlap considerably with XP-CS seems unjustified.

### Genes and phenotype

A recent report proposed that XP-CS may result from the retention of the NER intermediate TFIIH during cellular attempts at repairing UV and other bulky lesions [59]. During NER, a molecular complex called TFIIH wedges between DNA strands at the site of a lesion. The report proposed that this complex may be retained in all XP-CS cells, thereby inhibiting DNA synthesis, and creating a situation where DNA breaks can occur at sites of repair incisions.

The phenotypes resulting from mutations in the XP genes vary from uncomplicated XP to XP with neurological problems that may or may not be CS-like. There are also cases that are not easily classified, such as XP24BR, CO14TA, and CO107TA, the three XP-F patients described above. Additionally, two Finnish individuals with *XPG* mutations had CS features such as remarkably short stature, hearing loss, and bird-like faces, yet their intelligence was apparently normal and they had relatively few neurological problems at the ages of 22 and 34 [60]. Precise *XPG* mutations were not known in these individuals.

After reviewing mutations and case reports in XP-G patients, it appears that disease severity appears to be related to the mutated *XPG*’s DNA repair ability. Patients with complete loss of XPG DNA repair ability due to either missing domains, point mutations near the N-terminus, or truncation have the most severe symptoms [61]. Lehmann et al. mapped the essential amino acids for TFIIH interaction to residues 30 through 175. Patients with at least one allele with a missense mutation early in the reading frame had severe cases of XP-CS [47, 62], whereas those with missense mutations later in the reading frame tended to display the XP phenotype without characteristics of CS.

Harada et al. demonstrated that XPG null mice died soon after birth. This finding also suggests that viability may be predicated on the presence and partial functionality of XPG. Two other studies also found that the most severe cases of XP-CS in their cohort resulted from severely truncated XPG proteins that were suspected to be nonfunctional [24, 63], compared with a milder case in a compound heterozygous patient with one partially functional copy of the XPG gene. Again, these findings suggest that partial functionality in at least one allele may result in a milder phenotype.

Interestingly, Fassihi et al. found two patients with a homozygous single base pair mutation early in the reading frame but with a mild XP-CS phenotype [14]. Upon further sequence analysis, the point mutation was found to cause aberrant splice products, and potentially allows for occasional normal read through of the gene—resulting in protein that might retain enough stability and function to mitigate the patients’ symptoms. This also implies that partial protein function may be enough to alleviate the severity of disease seen in patients with similar mutations.

## Conclusions

Since the last review of XP-CS in 2001, the number of patients described in the literature has increased by a factor of almost five. The greater number of patients has allowed a more thorough analysis of XP-CS than was previously possible. A result is that much has been learned about the clinical manifestations of this disease. In addition, advances in clinical care based on lessons from both xeroderma pigmentosum and Cockayne syndrome have improved the ability to manage XP-CS as a combined condition. Much work remains to be done, however. Future published case histories would benefit the field by providing as much detail as possible about new XP-CS patients.

## Additional files

Additional file 1: **Tables S1–S3.** International designations, ethnic origins, and complementation groups of XP-CS patients analyzed in this review. (DOCX 31 kb)

Additional file 2: **Bibliography 1.** Bibliography of XP-CS patients analyzed in this review. (DOCX 76 kb)

Additional file 3: **Bibliography 2.** Bibliography of XP-F and XP-G patients identified in the literature. (DOCX 76 kb)
