# Supplementary material for: Xeroderma pigmentosum-Cockayne syndrome complex
Source: Orphanet J Rare Dis. 2017 Apr 4;12:65. doi: 10.1186/s13023-017-0616-2 (PMC5379700; doi:10.1186/s13023-017-0616-2)
Supplement: Supplementary file 1 — Tables S1–S3. International designations, ethnic origins, and complementation groups of XP-CS patients analyzed in this review. (DOCX 31 kb) [file 13023_2017_616_MOESM1_ESM.docx]

| **Number** | **Patient** | **Compl. group** | **Other designations** | **Source** |
| --- | --- | --- | --- | --- |
| 1 | CS1USAU | XP-F |  | Kashiyama |
| 2 | XPCS1BA | XP-B |  | Scott |
| 3 | XPCS1BD | XP-G |  | Thorel |
| 4 | XPCS1CD | XP-F |  | Kashiyama |
| 5 | XPCS1LV | XP-G | NF | Jaeken |
| 6 | XPCS1PV | XP-D |  | Theron |
| 7 | XPCS1RO | XP-G | 94RD27 | Hamel |
| 8 | XP1JI | XP-D |  | Fujimoto |
| 9 | XP1NE | XP-D |  | Fujimoto |
| 10 | XPCS2 | XP-D | XP-SC-8, XP-H | Moshell |
| 11 | XPCS2BA | XP-B |  | Scott |
| 12 | XPCS2LV | XP-G | BT | Jaeken |
| 13 | XPCS2RO | XP-D | 96RD362 | Graham |
| 14 | XP2BI | XP-G |  | Cheesbrough |
| 15 | XP3BR | XP-G |  | Arlett |
| 16 | XPCS4RO | XP-G |  | Zafeiriou |
| 17 | XP8BR | XP-D |  | Broughton |
| 18 | XP11BE | XP-B | Case 11, XPCS1 | Robbins |
| 19 | XP20BE | XP-G |  | Moriwaki |
| 20 | XP51RO | XP-F | XFE | Niedernhofer |
| 21 | XP55BR | XP-G |  | Fassihi |
| 22 | XP56BR | XP-G |  | Fassihi |
| 23 | XP72MA | XP-G |  | Schaefer |
| 24 | XP82DC | XP-G |  | Emmert |
| 25 | XP89MA | XP-D |  | Schaefer |
| 26 | XP96TA | XP-G |  | Emmert |
| 27 | XP104BR | XP-G |  | Fassihi |
| 28 | XPCS118LV | XP-D |  | Theron |
| 29 | XP131MA | XP-B |  | Bartenjev |
| 30 | XPCS142NH | XP-G |  | Falik-Z |
| 31 | XP165MA | XP-G |  | Schaefer |
| 32 | XP172MA | XP-G |  | Lehmann |
| 33 | XP183MA | XP-B |  | Oh |
| 34 | XP218BE | XP-G |  | Totonchy |
| 35 | COFS-05-135 | XP-D |  | Horibata |
| 36 | Brother_05135 | XP-D |  | Horibata |
| 37 | COFS-Chiba1 | XP-D |  | Horibata |
| 38 | Brother_Chiba1 | XP-D |  | Horibata |
| 39 | Hijazi_1 | XP-G |  | Hijazi |
| 40 | Hijazi_2 | XP-G |  | Hijazi |
| 41 | Hijazi_3 | XP-G |  | Hijazi |
| 42 | Kondo_male | XP-D |  | Kondo |
| 43 | Singh_female | Unknown |  | Singh |

**Table S1. XP-CS patients.** The table shows patient designations, alternate designations used in the literature, and the surname of the first author of the primary reference for each patient. This table does not include two potential adult-onset CS patients (CO14TA & CO107TA), as their diagnosis had not been confirmed.

| National origin | Number |
| --- | --- |
| Afghanistan | 1 |
| Belgium | 3 |
| Canada | 1 |
| Germany | 4 |
| Greece | 1 |
| India | 1 |
| Israel | 2 |
| Japan | 6 |
| Morocco | 1 |
| Pakistan | 1 |
| Slovenia | 1 |
| Somalia | 2 |
| Spain | 2 |
| Sudan | 3 |
| Switzerland | 2 |
| Turkey | 1 |
| UK | 5 |
| USA | 6 |
| **Total** | **43** |

**Table S2. National origin of XP-CS patients.** XP-CS patients lived in or had parental origins in 18 different countries on four continents (Africa, Asia, Europe, and North America).

| **Race/ethnic group** | **XP-B** | **XP-D** | **XP-F** | **XP-G** |
| --- | --- | --- | --- | --- |
| Caucasian | 5 | 5 | 3 | 8 |
| Asian/Japan | - | 6 | - | - |
| Asian/Pakistan | - | - | 1 | - |
| Arab | - | - | - | 5 |
| African or African American | - | - | - | 3 |
| **Total** | **5** | **11** | **4** | **16** |

**Table S3. Complementation group by race or ethnic group.** Data for complementation group and ethnic group or race was available for 36 patients.

**Supplemental figure. XP-G patients by phenotype.** A total of 33 people with mutations in XP-G were found in the literature. We believe that this figure represents all or nearly all XP-G patients reported to date. Of this group, 58% had XP-CS, 28% had XP without neurological abnormalities, and 14% had XP with neurological abnormalities that were not CS-like.
