# Supplementary material for: Xeroderma pigmentosum-Cockayne syndrome complex
Source: Orphanet J Rare Dis. 2017 Apr 4;12:65. doi: 10.1186/s13023-017-0616-2 (PMC5379700; doi:10.1186/s13023-017-0616-2)
Supplement: Supplementary file 2 — Bibliography 1. Bibliography of XP-CS patients analyzed in this review. (DOCX 76 kb) [file 13023_2017_616_MOESM2_ESM.docx]

**Additional file 2: Supplemental Bibliography 1: published case histories of XP-CS patients**

Patient designations are boldfaced. Alternative designations are in parentheses. References for cases of XP-G patients without CS are in Supplemental Bibliography 2. This list does not include patients CO14TA and CO107TA, as their diagnoses were not confirmed (see Supplemental Bibliography 2).

- **XP-B (5 patients): XPCS1BA** [[1](#_ENREF_1)], **XPCS2BA** [[1](#_ENREF_1)], **XP11BE (Case 11, XPCS1)** [[2-4](#_ENREF_2)], **XP131MA**[[5](#_ENREF_5)], **XP183MA**[[6](#_ENREF_6)].
- **XP-D (13 patients): XP1J1**[[7](#_ENREF_7)], **XP1NE** [[7](#_ENREF_7)], **XPCS1PV** [[8](#_ENREF_8)], **XPCS2 (XP-SC-8)** [[9](#_ENREF_9)], **XPCS2RO (96RD362)** [[10](#_ENREF_10)], **XP8BR** [[11](#_ENREF_11)], **XP89MA** [[12](#_ENREF_12)], **XPCS118LV** [[8](#_ENREF_8)], **COFS-05-135** [[13](#_ENREF_13)], **Brother of** **COFS-05-135** [[13](#_ENREF_13)], **COFS-Chiba1** [[13](#_ENREF_13)], **Brother of COFS-Chiba1** [[13](#_ENREF_13)], **Kondo-male** [[14](#_ENREF_14)]
- **XP-G (21 patients): XPCS1BD** [[15](#_ENREF_15)], **XPCS1LV (NF)** [[16](#_ENREF_16)], **XPCS1RO (94RD27)** [[17](#_ENREF_17)], **XP2BI [**[**18**](#_ENREF_18)**]**, **XPCS2LV (BT)** [[16](#_ENREF_16)], **XP3BR** [[19](#_ENREF_19)], **XPCS4RO** [[20](#_ENREF_20)], **XP55BR[**[**21**](#_ENREF_21)**]**, **XP56BR**[[21](#_ENREF_21)], **XP20BE**[[22-24](#_ENREF_22)], **XP72MA**[[25](#_ENREF_25)], **XP82DC** [[26](#_ENREF_26)], **XP165MA** [[25](#_ENREF_25)], **XP96TA**[[26](#_ENREF_26)], **XP104BR**[[21](#_ENREF_21)],**XPCS142NH**[[27](#_ENREF_27)], **XP172MA**[[28](#_ENREF_28)], **XP218BE [**[**23**](#_ENREF_23)**]**, **Patient S1 [**[**29**](#_ENREF_29)**]**, **Patient S2 [**[**29**](#_ENREF_29)**]**, **Patient S3** [[29](#_ENREF_29)].
- **XP-F (3 patients): CS1USAU [**[**30**](#_ENREF_30)**]**, **XPCS1CD [**[**30**](#_ENREF_30)**]**, **XP51RO** **(XFE)** [[31](#_ENREF_31), [32](#_ENREF_32)].
- **Complementation group not known (1 patient): Singh-female** [[33](#_ENREF_33)].

**References**

1. Scott RJ, Itin P, Kleijer WJ, Kolb K, Arlett C, Muller H: **Xeroderma pigmentosum-Cockayne syndrome complex in two patients: absence of skin tumors despite severe deficiency of DNA excision repair**. *J Am Acad Dermatol* 1993, **29**(5 Pt 2):883-889.

2. Robbins JH, Kraemer KH, Lutzner MA, Festoff BW, Coon HG: **Xeroderma pigmentosum. An inherited diseases with sun sensitivity, multiple cutaneous neoplasms, and abnormal DNA repair**. *Ann Intern Med* 1974, **80**(2):221-248.

3. Brumback RA, Yoder FW, Andrews AD, Peck GL, Robbins JH: **Normal pressure hydrocephalus. Recognition and relationship to neurological abnormalities in Cockayne's syndrome**. *Arch Neurol* 1978, **35**(6):337-345.

4. Noojin RO: **Xeroderma pigmentosum treated with oral methoxsalen**. *Arch Dermatol* 1965, **92**(4):422-423.

5. Bartenjev I, Butina MR, Potocnik M: **Rare case of Cockayne syndrome with xeroderma pigmentosum**. *Acta Derm Venereol* 2000, **80**(3):213-214.

6. Oh KS, Khan SG, Jaspers NG, Raams A, Ueda T, Lehmann A, Friedmann PS, Emmert S, Gratchev A, Lachlan K *et al*: **Phenotypic heterogeneity in the XPB DNA helicase gene (ERCC3): xeroderma pigmentosum without and with Cockayne syndrome**. *Hum Mutat* 2006, **27**(11):1092-1103.

7. Fujimoto M, Leech SN, Theron T, Mori M, Fawcett H, Botta E, Nozaki Y, Yamagata T, Moriwaki S, Stefanini M *et al*: **Two new XPD patients compound heterozygous for the same mutation demonstrate diverse clinical features**. *J Invest Dermatol* 2005, **125**(1):86-92.

8. Theron T, Fousteri MI, Volker M, Harries LW, Botta E, Stefanini M, Fujimoto M, Andressoo JO, Mitchell J, Jaspers NG *et al*: **Transcription-associated breaks in xeroderma pigmentosum group D cells from patients with combined features of xeroderma pigmentosum and Cockayne syndrome**. *Mol Cell Biol* 2005, **25**(18):8368-8378.

9. Moshell AG, MB; Lutzner, MA; Coon, HG; Barrett, SF; Dupuy, JM; Robbins, JH. : **A new patient with both xeroderma pigmentosum and Cockayne syndrome establishes the new xeroderma pigmentosum complementation group H.** In: *Cellular Responses to DNA Damage.* Edited by Friedberg EB, BA. New York: Alan R Liss; 1983: 209-213.

10. Graham JM, Jr., Anyane-Yeboa K, Raams A, Appeldoorn E, Kleijer WJ, Garritsen VH, Busch D, Edersheim TG, Jaspers NG: **Cerebro-oculo-facio-skeletal syndrome with a nucleotide excision-repair defect and a mutated XPD gene, with prenatal diagnosis in a triplet pregnancy**. *Am J Hum Genet* 2001, **69**(2):291-300.

11. Broughton BC, Thompson AF, Harcourt SA, Vermeulen W, Hoeijmakers JH, Botta E, Stefanini M, King MD, Weber CA, Cole J *et al*: **Molecular and cellular analysis of the DNA repair defect in a patient in xeroderma pigmentosum complementation group D who has the clinical features of xeroderma pigmentosum and Cockayne syndrome**. *Am J Hum Genet* 1995, **56**(1):167-174.

12. Schafer A, Gratchev A, Seebode C, Hofmann L, Schubert S, Laspe P, Apel A, Ohlenbusch A, Tzvetkov M, Weishaupt C *et al*: **Functional and molecular genetic analyses of nine newly identified XPD-deficient patients reveal a novel mutation resulting in TTD as well as in XP/CS complex phenotypes**. *Exp Dermatol* 2013, **22**(7):486-489.

13. Horibata K, Kono S, Ishigami C, Zhang X, Aizawa M, Kako Y, Ishii T, Kosaki R, Saijo M, Tanaka K: **Constructive rescue of TFIIH instability by an alternative isoform of XPD derived from a mutated XPD allele in mild but not severe XP-D/CS**. *J Hum Genet* 2015, **60**(5):259-265.

14. Kondo D, Noguchi A, Tamura H, Tsuchida S, Takahashi I, Kubota H, Yano T, Oyama C, Sawaishi Y, Moriwaki S *et al*: **Elevated Urinary Levels of 8-Hydroxy-2'-deoxyguanosine in a Japanese Child of Xeroderma Pigmentosum/Cockayne Syndrome Complex with Infantile Onset of Nephrotic Syndrome**. *Tohoku J Exp Med* 2016, **239**(3):231-235.

15. Thorel F, Constantinou A, Dunand-Sauthier I, Nouspikel T, Lalle P, Raams A, Jaspers NG, Vermeulen W, Shivji MK, Wood RD *et al*: **Definition of a short region of XPG necessary for TFIIH interaction and stable recruitment to sites of UV damage**. *Mol Cell Biol* 2004, **24**(24):10670-10680.

16. Jaeken J, Klocker H, Schwaiger H, Bellmann R, Hirsch-Kauffmann M, Schweiger M: **Clinical and biochemical studies in three patients with severe early infantile Cockayne syndrome**. *Hum Genet* 1989, **83**(4):339-346.

17. Hamel BC, Raams A, Schuitema-Dijkstra AR, Simons P, van der Burgt I, Jaspers NG, Kleijer WJ: **Xeroderma pigmentosum--Cockayne syndrome complex: a further case**. *J Med Genet* 1996, **33**(7):607-610.

18. Cheesbrough MJ: **Xeroderma pigmentosum--a unique variant with neurological involvement**. *Br J Dermatol* 1978, **99**(Suppl 16):61.

19. Arlett CF, Harcourt SA, Lehmann AR, Stevens S, Ferguson-Smith MA, Morley WN: **Studies on a new case of xeroderma pigmentosum (XP3BR) from complementation group G with cellular sensitivity to ionizing radiation**. *Carcinogenesis* 1980, **1**(9):745-751.

20. Zafeiriou DI, Thorel F, Andreou A, Kleijer WJ, Raams A, Garritsen VH, Gombakis N, Jaspers NG, Clarkson SG: **Xeroderma pigmentosum group G with severe neurological involvement and features of Cockayne syndrome in infancy**. *Pediatr Res* 2001, **49**(3):407-412.

21. Fassihi H, Sethi M, Fawcett H, Wing J, Chandler N, Mohammed S, Craythorne E, Morley AM, Lim R, Turner S *et al*: **Deep phenotyping of 89 xeroderma pigmentosum patients reveals unexpected heterogeneity dependent on the precise molecular defect**. *Proc Natl Acad Sci U S A* 2016, **113**(9):E1236-1245.

22. Moriwaki S, Stefanini M, Lehmann AR, Hoeijmakers JH, Robbins JH, Rapin I, Botta E, Tanganelli B, Vermeulen W, Broughton BC *et al*: **DNA repair and ultraviolet mutagenesis in cells from a new patient with xeroderma pigmentosum group G and cockayne syndrome resemble xeroderma pigmentosum cells**. *J Invest Dermatol* 1996, **107**(4):647-653.

23. Lindenbaum Y, Dickson D, Rosenbaum P, Kraemer K, Robbins I, Rapin I: **Xeroderma pigmentosum/cockayne syndrome complex: first neuropathological study and review of eight other cases**. *Eur J Paediatr Neurol* 2001, **5**(6):225-242.

24. Rapin I, Lindenbaum Y, Dickson DW, Kraemer KH, Robbins JH: **Cockayne syndrome and xeroderma pigmentosum**. *Neurology* 2000, **55**(10):1442-1449.

25. Schafer A, Schubert S, Gratchev A, Seebode C, Apel A, Laspe P, Hofmann L, Ohlenbusch A, Mori T, Kobayashi N *et al*: **Characterization of three XPG-defective patients identifies three missense mutations that impair repair and transcription**. *J Invest Dermatol* 2013, **133**(7):1841-1849.

26. Emmert S, Slor H, Busch DB, Batko S, Albert RB, Coleman D, Khan SG, Abu-Libdeh B, DiGiovanna JJ, Cunningham BB *et al*: **Relationship of neurologic degeneration to genotype in three xeroderma pigmentosum group G patients**. *J Invest Dermatol* 2002, **118**(6):972-982.

27. Falik-Zaccai TC, Erel-Segal R, Horev L, Bitterman-Deutsch O, Koka S, Chaim S, Keren Z, Kalfon L, Gross B, Segal Z *et al*: **A novel XPD mutation in a compound heterozygote; the mutation in the second allele is present in three homozygous patients with mild sun sensitivity**. *Environ Mol Mutagen* 2012, **53**(7):505-514.

28. Lehmann J, Schubert S, Schafer A, Apel A, Laspe P, Schiller S, Ohlenbusch A, Gratchev A, Emmert S: **An unusual mutation in the XPG gene leads to an internal in-frame deletion and a XP/CS complex phenotype**. *Br J Dermatol* 2014, **171**(4):903-905.

29. Hijazi H, Salih MA, Hamad MH, Hassan HH, Salih SB, Mohamed KA, Mukhtar MM, Karrar ZA, Ansari S, Ibrahim N *et al*: **Pellagra-like condition is xeroderma pigmentosum/Cockayne syndrome complex and niacin confers clinical benefit**. *Clin Genet* 2015, **87**(1):56-61.

30. Kashiyama K, Nakazawa Y, Pilz DT, Guo C, Shimada M, Sasaki K, Fawcett H, Wing JF, Lewin SO, Carr L *et al*: **Malfunction of nuclease ERCC1-XPF results in diverse clinical manifestations and causes Cockayne syndrome, xeroderma pigmentosum, and Fanconi anemia**. *Am J Hum Genet* 2013, **92**(5):807-819.

31. Niedernhofer LJ, Garinis GA, Raams A, Lalai AS, Robinson AR, Appeldoorn E, Odijk H, Oostendorp R, Ahmad A, van Leeuwen W *et al*: **A new progeroid syndrome reveals that genotoxic stress suppresses the somatotroph axis**. *Nature* 2006, **444**(7122):1038-1043.

32. Brooks BP, Thompson AH, Bishop RJ, Clayton JA, Chan CC, Tsilou ET, Zein WM, Tamura D, Khan SG, Ueda T *et al*: **Ocular manifestations of xeroderma pigmentosum: long-term follow-up highlights the role of DNA repair in protection from sun damage**. *Ophthalmology* 2013, **120**(7):1324-1336.

33. Singh UR, Asif S, Kommu PP, D'Souza P: **Cockayne syndrome-xeroderma pigmentosum complex with demyelination: A rare association**. *Indian J Hum Genet* 2012, **18**(1):125-126.
