# Supplementary material for: Xeroderma pigmentosum-Cockayne syndrome complex
Source: Orphanet J Rare Dis. 2017 Apr 4;12:65. doi: 10.1186/s13023-017-0616-2 (PMC5379700; doi:10.1186/s13023-017-0616-2)
Supplement: Supplementary file 3 — Bibliography 2. Bibliography of XP-F and XP-G patients identified in the literature. (DOCX 76 kb) [file 13023_2017_616_MOESM3_ESM.docx]

**Additional file 3:Supplemental Bibliography 2: published case histories of XP-F & XP-G patients**

This list contains information on XP-F and -G patients with XP or XP neurological disease. XP-F or -G patients with CS are listed in Supplemental Biography 1. This list is included here for the purposes of completeness. We have endeavored to find all patients in these complementation groups, but may have missed some. If patients were not given international designations, they are identified here as AuthorName_NumberSex.

Creating a list of XP-D patients was not possible due to large patient numbers. References for all 10 XP-B patients are cited in the main text.

- **XP-F (43 patients without confirmed CS):** **XP1TS** [[1](#_ENREF_1)], **XP2YO** [[2](#_ENREF_2), [3](#_ENREF_3)], **XP3YO** [[2](#_ENREF_2), [3](#_ENREF_3)], **XP7KA** [[2](#_ENREF_2)], **XP7NE** [[4](#_ENREF_4)], **XP13NA** [[2](#_ENREF_2)], **XP14PF** [[5](#_ENREF_5)], **XP23OS** [[6](#_ENREF_6)], **XP24BR** [[7](#_ENREF_7)], **XP24KY** [[1](#_ENREF_1)], **XP25KO** [[8](#_ENREF_8), [9](#_ENREF_9)], **XP26BR** [[10](#_ENREF_10)], **XP27KO** [[8](#_ENREF_8), [9](#_ENREF_9)], **XP28KO** [[8](#_ENREF_8), [9](#_ENREF_9)], **XP29MA** [[11](#_ENREF_11)], **XP30MA** [[11](#_ENREF_11)], **XP32BR** [[7](#_ENREF_7)], **XP38KO** [[8](#_ENREF_8), [9](#_ENREF_9)], **XP41KO** [[2](#_ENREF_2)], **XP42RO** [[12](#_ENREF_12)], **XP46KO** [[9](#_ENREF_9)], **XP48DC** [[13](#_ENREF_13)], **XP62RO** [[10](#_ENREF_10)], **XP72BR** [[7](#_ENREF_7)], **XP90TO** [[2](#_ENREF_2)], **XP90TOA** [[2](#_ENREF_2)], **XP101OS** [[14](#_ENREF_14)], **XP107TO** [[2](#_ENREF_2), [15](#_ENREF_15)], **XP126LO** [[16](#_ENREF_16)], **AS871** [[10](#_ENREF_10)], **Asai_1F** [[17](#_ENREF_17)], **Carré_1F** [[18](#_ENREF_18)], **Carré_2M** [[18](#_ENREF_18)], **CO14TA** (may have had adult-onset CS) [[13](#_ENREF_13)], **CO107TA** (may have had adult-onset CS) [[13](#_ENREF_13)], **FA104** (had Fanconi anemia) [[19](#_ENREF_19)], **Ito_1F** [[20](#_ENREF_20)], **Kato_1F** [[21](#_ENREF_21)], **Kps6** [[22](#_ENREF_22)], **Minamino_1F** [[23](#_ENREF_23), [24](#_ENREF_24)], **MNHN** [[25](#_ENREF_25)], **Tofuku_1F** [[24](#_ENREF_24)], **Yasue_1F** [[26](#_ENREF_26)].
- **XP-G (21 patients without CS): XP1HF** [[27](#_ENREF_27)], **XP01RJ** [[28](#_ENREF_28)], **XP02RJ** [[28](#_ENREF_28)], **XP3HM** [[29](#_ENREF_29)], **XP12PF** [[5](#_ENREF_5)], **XP13PF** [[5](#_ENREF_5)], **XP31KO** [[30](#_ENREF_30)], **XP34BR** [[7](#_ENREF_7)], **XP40GO** (phenotype not known) [[31](#_ENREF_31)], **XP52HM** [[29](#_ENREF_29)], **XP65BE** [[32](#_ENREF_32)], **XP101BR** [[7](#_ENREF_7)], **XP118BR** [[7](#_ENREF_7)], **XP120BR** [[7](#_ENREF_7)], **XP124LO** [[33](#_ENREF_33)], **XP125LO** [[33](#_ENREF_33)], **XP915** [[34](#_ENREF_34)], **XP918** [[34](#_ENREF_34)], **Anttinen_16** [[27](#_ENREF_27)], **Wang_1F** [[35](#_ENREF_35)], **Zhang_5F** [[36](#_ENREF_36)].

1. Matsumura Y, Nishigori C, Yagi T, Imamura S, Takebe H: **Characterization of molecular defects in xeroderma pigmentosum group F in relation to its clinically mild symptoms**. *Hum Mol Genet* 1998, **7**(6):969-974.

2. Kondo S, Miyamoto C, H. KC, Satoh Y, Fujiwara Y, Seki S, Hidekazu K: **Sib patients bearing basaliomas in xeroderma pigmentosum complementation group F.** *Nishi Nihon Hifuka* 1990, **52**(2):279-284.

3. Takebe H, Nikaido O, Ishizaki K: **Genetic aspect of xeroderma pigmentosum and other cancer-prone diseases.** In: *Genetic and Environmental Factors in Experimental and Human Canc.* Edited by Gelboin HV, Kikin TnMHGK. Tokyo: Japan Science Society Press; 1980: 259-270.

4. Arlett CF, Harcourt SA, Cole J, Green MH, Anstey AV: **A comparison of the response of unstimulated and stimulated T-lymphocytes and fibroblasts from normal, xeroderma pigmentosum and trichothiodystrophy donors to the lethal action of UV-C**. *Mutat Res* 1992, **273**(2):127-135.

5. Zhou EY, Wang H, Lin Z, Xu G, Ma Z, Zhao J, Feng C, Duo L, Yin J, Yang Y: **Clinical and molecular epidemiological study of xeroderma pigmentosum in China: A case series of 19 patients**. *J Dermatol* 2017, **44**(1):71-75.

6. Arase S, Kozuka T, Tanaka K, Ikenaga M, Takebe H: **A sixth complementation group in xeroderma pigmentosum**. *Mutat Res* 1979, **59**(1):143-146.

7. Fassihi H, Sethi M, Fawcett H, Wing J, Chandler N, Mohammed S, Craythorne E, Morley AM, Lim R, Turner S *et al*: **Deep phenotyping of 89 xeroderma pigmentosum patients reveals unexpected heterogeneity dependent on the precise molecular defect**. *Proc Natl Acad Sci U S A* 2016, **113**(9):E1236-1245.

8. Fujiwara Y, Ichihashi M, Uehara Y, Matsumoto A, Yamamoto Y, Kano Y, Tanakura Y: **Xeroderma pigmentosum groups C and F: additional assignments and a review of the subjects in Japan**. *J Radiat Res* 1985, **26**(4):443-449.

9. Yamamura K, Ichihashi M, Hiramoto T, Ogoshi M, Nishioka K, Fujiwara Y: **Clinical and photobiological characteristics of xeroderma pigmentosum complementation group F: a review of cases from Japan**. *Br J Dermatol* 1989, **121**(4):471-480.

10. Ahmad A, Enzlin JH, Bhagwat NR, Wijgers N, Raams A, Appledoorn E, Theil AF, JH JH, Vermeulen W, NG JJ *et al*: **Mislocalization of XPF-ERCC1 nuclease contributes to reduced DNA repair in XP-F patients**. *PLoS Genet* 2010, **6**(3):e1000871.

11. Thielmann HW, Fischer E, Dzarlieva RT, Komitowski D, Popanda O, Edler L: **Spontaneous in vitro malignant transformation in a xeroderma pigmentosum fibroblast line**. *Int J Cancer* 1983, **31**(6):687-700.

12. Sijbers AM, van Voorst Vader PC, Snoek JW, Raams A, Jaspers NG, Kleijer WJ: **Homozygous R788W point mutation in the XPF gene of a patient with xeroderma pigmentosum and late-onset neurologic disease**. *J Invest Dermatol* 1998, **110**(5):832-836.

13. Imoto K, Slor H, Orgal D, Khan SG, Busch DB, Nadem C, Ueda T, Gadoth N, Jaspers NJ, Kraemer K: **Xeroderma pigmentosum group F patients with late onset neurological disease. (Conference abstract)**. *J Invest Dermatol* 2005, **124**(4):A78.

14. Nishigori C, Ishizaki K, Takebe H, Imamura S, Hayakawa M: **A case of xeroderma pigmentosum group F with late onset of clinical symptoms**. *Arch Dermatol* 1986, **122**(5):510-511.

15. Nishigori C, Fujisawa H, Uyeno K, Kawaguchi T, Takebe H: **Xeroderma pigmentosum patients belonging to complementation group F and efficient liquid-holding recovery of ultraviolet damage**. *Photodermatol Photoimmunol Photomed* 1991, **8**(4):146-150.

16. Norris PG, Hawk JL, Avery JA, Giannelli F: **Xeroderma pigmentosum complementation group F in a non-Japanese patient**. *J Am Acad Dermatol* 1988, **18**(5 Pt 2):1185-1188.

17. Asai M, Koike Y, Tomimura S, Takenaka M, Utani A: **Long-term Follow-up of a Case of Xeroderma Pigmentosum Complementation Group F**. *Nishi Nihon Hifuka* 2013, **75**(6):508-510.

18. Carré G, Marelli C, Geny C, Rezvani H, Koenig M, Anheim M, Tranchant C: **Xeroderma pigmentosum de type F : une cause rare d’ataxie cérébelleuse récessive**. *Revue Neurologique* 2016, **172, Supplement 1**:A125.

19. Bogliolo M, Schuster B, Stoepker C, Derkunt B, Su Y, Raams A, Trujillo JP, Minguillon J, Ramirez MJ, Pujol R *et al*: **Mutations in ERCC4, encoding the DNA-repair endonuclease XPF, cause Fanconi anemia**. *Am J Hum Genet* 2013, **92**(5):800-806.

20. Ito T, Watanabe H, Yamaizumi M, Ono T: **Pigmentary xeroderma group F group complicated with basal cell carcinoma. (Conference abstract)**. *Jpn J Dermatol* 1995(105):424.

21. Kato Y, Muro Y, Yasue T, Matsumoto Y, Ohashi MA-C-FaiapwxpiJJJD: **Anti-CENP-F antibody in a patient with xeroderma pigmentosum.** *Jpn J Dermatol* 2000, **110**:301-306.

22. Itoh T, Watanabe H, Yamaizumi M, Ono T: **A young woman with xeroderma pigmentosum complementation group F and a morphoeic basal cell carcinoma**. *Br J Dermatol* 1995, **132**(1):122-127.

23. Minamino Y, Fujisawa H: **A case of xeroderma pigmentosum group F. (Conference abstract)**. *Jpn J Dermatol* 2002, **112**:866.

24. Tofuku Y, Nobeyama Y, Kamide R, Moriwaki S, Nakagawa H: **Xeroderma pigmentosum complementation group F: Report of a case and review of Japanese patients**. *J Dermatol* 2015, **42**(9):897-899.

25. Moriwaki S, Nishigori C, Imamura S, Yagi T, Takahashi C, Fujimoto N, Takebe H: **A case of xeroderma pigmentosum complementation group F with neurological abnormalities**. *Br J Dermatol* 1993, **128**(1):91-94.

26. Yasue T, Hiraiwa A, Matsumoto Y, Ohashi M, Torii S, Matsumura Y, Takebe H: **A case of xeroderma pigmentosum group F. (Conference abstract)**. *Jpn J Dermatol* 1998, **108**:883.

27. Anttinen A, Koulu L, Nikoskelainen E, Portin R, Kurki T, Erkinjuntti M, Jaspers NG, Raams A, Green MH, Lehmann AR *et al*: **Neurological symptoms and natural course of xeroderma pigmentosum**. *Brain* 2008, **131**(Pt 8):1979-1989.

28. Soltys DT, Rocha CR, Lerner LK, de Souza TA, Munford V, Cabral F, Nardo T, Stefanini M, Sarasin A, Cabral-Neto JB *et al*: **Novel XPG (ERCC5) mutations affect DNA repair and cell survival after ultraviolet but not oxidative stress**. *Hum Mutat* 2013, **34**(3):481-489.

29. Moriwaki S, Takigawa M, Igarashi N, Nagai Y, Amano H, Ishikawa O, Khan SG, Kraemer KH: **Xeroderma pigmentosum complementation group G patient with a novel homozygous missense mutation and no neurological abnormalities**. *Exp Dermatol* 2012, **21**(4):304-307.

30. Ichihashi M, Fujiwara Y, Uehara Y, Matsumoto A: **A mild form of xeroderma pigmentosum assigned to complementation group G and its repair heterogeneity**. *J Invest Dermatol* 1985, **85**(3):284-287.

31. Schafer A, Schubert S, Gratchev A, Seebode C, Apel A, Laspe P, Hofmann L, Ohlenbusch A, Mori T, Kobayashi N *et al*: **Characterization of three XPG-defective patients identifies three missense mutations that impair repair and transcription**. *J Invest Dermatol* 2013, **133**(7):1841-1849.

32. Emmert S, Slor H, Busch DB, Batko S, Albert RB, Coleman D, Khan SG, Abu-Libdeh B, DiGiovanna JJ, Cunningham BB *et al*: **Relationship of neurologic degeneration to genotype in three xeroderma pigmentosum group G patients**. *J Invest Dermatol* 2002, **118**(6):972-982.

33. Norris PG, Hawk JL, Avery JA, Giannelli F: **Xeroderma pigmentosum complementation group G--report of two cases**. *Br J Dermatol* 1987, **116**(6):861-866.

34. Sun Z, Zhang J, Guo Y, Ni C, Liang J, Cheng R, Li M, Yao Z: **Genotype-phenotype correlation of xeroderma pigmentosum in a Chinese Han population**. *Br J Dermatol* 2015, **172**(4):1096-1102.

35. Wang T, Xu CC, Zhou XP, Lee JJ, Shen J, Lian BQ, Liu YH, Lian CG: **Novel germline ERCC5 mutations identified in a xeroderma pigmentosum complementation group G pedigree**. *JAAD Case Rep* 2015, **1**(2):66-70.

36. Zhang J, Cheng R, Yu X, Sun Z, Li M, Yao Z: **Expansion of the genotypic and phenotypic spectrum of xeroderma pigmentosum in Chinese population**. *Photodermatol Photoimmunol Photomed* 2016.
